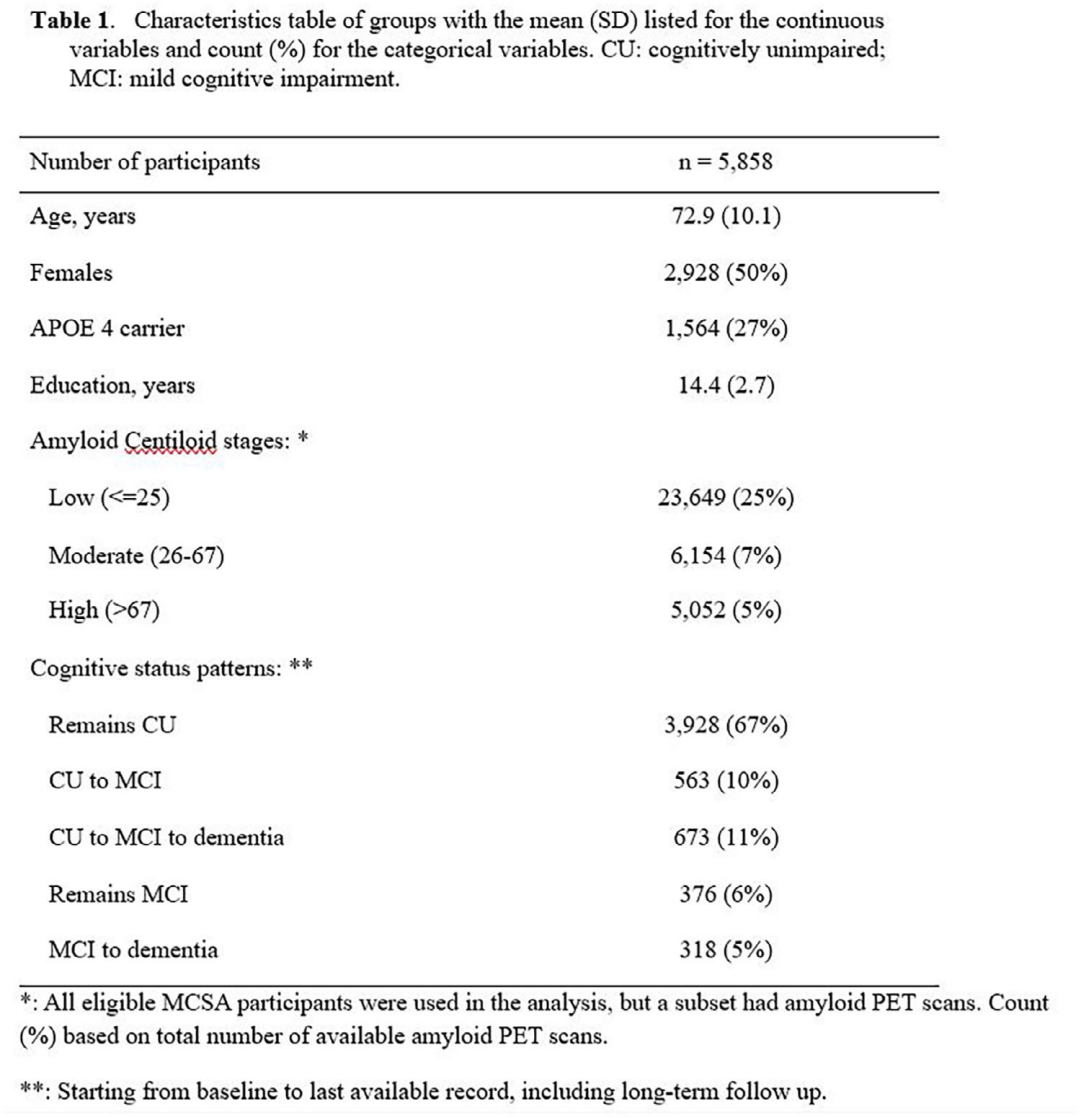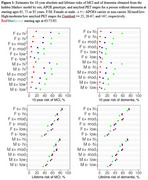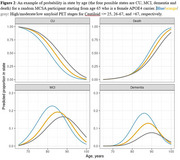# Absolute risk of cognitive impairment by AD amyloid stages

**DOI:** 10.1002/alz70862_109822

**Published:** 2025-12-23

**Authors:** Mingzhao Hu

**Affiliations:** ^1^ Department of Quantitative Health Sciences, Mayo Clinic, Rochester, MN USA

## Abstract

**Background:**

Despite advances in Alzheimer’s disease (AD) research, limited information exists regarding the absolute risk of mild cognitive impairment (MCI) in cognitively unimpaired (CU) individuals with abnormal AD biomarkers, particularly when accounting for competing risks of death. This knowledge gap is critical as the field addresses disease‐modifying treatments targeting CU individuals with preclinical AD. We focus on the predictive value of AD amyloid stages defined by amyloid PET Centiloid values.

**Methods:**

We included 5,858 participants from the Mayo Clinic Study of Aging (MCSA) to evaluate AD amyloid stage as a predictor of clinical progression to MCI or dementia. The data includes long‐term follow‐up information on death and dementia beyond active study participation, which mitigates potential bias due to dropout. We predicted 10‐year and lifetime risks of MCI and dementia, accounting for the competing risks of death, given amyloid PET stages, sex, APOE4 status, and baseline age. Results are based on a hidden Markov model.

**Results:**

AD amyloid staging based on amyloid PET Centiloid values was the strongest predictor of lifetime risk for MCI or for dementia (Figure 1). Higher Centiloid levels amplified age effects on the risk of MCI, whereas for dementia, amyloid stage effects surpassed age effects. For 10‐year risk, age was the dominant factor, whereas for lifetime risk, amyloid stages had a greater influence. While smaller in magnitude than the effects of amyloid stages and age, APOE4 carrier status and (on average) male sex were also associated with increased risk of MCI and dementia. For a female APOE4 carrier at age 65, lifetime risk was 41%/61%/76% for low/moderate/high amyloid PET levels. The 10‐year absolute risk for a female APOE4 carrier with high amyloid PET was 4%/29%/58% when assessed at starting ages 65, 75, and 85.

**Conclusions:**

AD amyloid staging is a critical predictor of lifetime risk for cognitive impairment, underscoring its importance in guiding treatment decisions if interventions are approved for preclinical AD in the future. Effective patient evaluation should focus on biomarker‐based amyloid staging rather than simple binary biomarker classification, with careful consideration of age, sex, and APOE4 status to ensure a comprehensive benefit‐risk assessment for future therapeutic strategies.